# Chemoproteomic analysis reveals RECQL4 as a mediator of nitroalkene-dependent double-strand break repair inhibition in cancer.

**DOI:** 10.21203/rs.3.rs-6141403/v1

**Published:** 2025-03-26

**Authors:** Dennis C Braden, Mostafa A.L. Adbel-Salam, Alparslan Asan, John Skoko, Huiming Lu, Thomas P Conrads, Bruce A Freeman, Francisco J Schopfer, Ishu Saini, Jochen Kuper, Caroline Kisker, Apoorva Uboveja, Naveen K Tangudu, Katherine M Aird, Anthony J Davis, Carola A Neumann

**Affiliations:** 1 Department of Pharmacology and Chemical Biology, University of Pittsburgh, Pittsburgh, PA, 15261, USA; Women’s Cancer Research Center, University of Pittsburgh Cancer Institute, Pittsburgh, PA, 15213, USA; UPMC Hillman Cancer Center, Pittsburgh, PA, 15213, USA;; 2 Department of Radiation Oncology, UT Southwestern Medical Center, Dallas, TX75390, USA;; 3 Rudolf-Virchow-Zentrum-Center for Integrative and Translational Bioimaging Institute for Structural Biology, University of Würzburg, Würzburg, Germany.; 4 Department of Pharmacology and Chemical Biology, School of Medicine, University of Pittsburgh, Pittsburgh, PA, USA; Pittsburgh Heart, Lung, Blood, and Vascular Medicine Institute (VMI), University of Pittsburgh, Pittsburgh, PA, USA; Pittsburgh Liver Research Center (PLRC), University of Pittsburgh, Pittsburgh, PA, USA; Center for Metabolism and Mitochondrial Medicine (C3M) University of Pittsburgh, Pittsburgh, PA, USA;; 5 Gynecologic Cancer Center of Excellence, Department of Gynecologic Surgery and Obstetrics, Uniformed Services University of the Health Sciences, Walter Reed National Military Medical Center, Bethesda, MD 20889, USA; Women’s Health Integrated Research Center, Women’s Service Line, Inova Health System, Annandale, VA 22003, United States; Murtha Cancer Center Research Program, Department of Surgery, Uniformed Services University of the Health Sciences, Walter Reed National Military Medical Center, Bethesda, MD 20889, USA.

## Abstract

Nitroalkenes are endogenous products generated by the metabolism of unsaturated fatty acids. They are generated under oxidative stress conditions, mediating important anti-inflammatory signaling activities through covalent modification of protein cysteine thiols. Despite being cytoprotective in benign tissue, nitroalkenes display single-agent anti-proliferative activity in breast cancer cells and sensitize them to multiple DNA-damaging agents. Initial mechanistic evidence suggested that nitroalkene anti-cancer activities are partially mediated by inhibition of homologous recombination (HR) through the recombinase RAD51 at Cys319. However, nitroalkenes are multi-target agents, and thus, it is likely that other important DNA repair targets beyond RAD51 are modified by nitroalkenes, contributing to their anti-cancer effects. We, therefore, conducted a global proteomics analysis to address this question. This analysis led to the identification of the recQ helicase RECQL4 with a nitro-alkylation at Cys1052. This modification was further confirmed by click chemistry-based chemoproteomics and determined to be DNA damage-dependent. Functional analyses demonstrated that nitroalkene modification inhibits RECQL4 ATP-dependent helicase activity and disrupts DSB end resection and downstream homology-dependent repair. Furthermore, experiments with C1052S mutant RECQL4 revealed that RECQL4 is a major mediator of nitroalkene effects on end resection, DSB formation, and repair. The evidence presented here denotes RECQL4 as an important nitroalkene target conferring DSB repair inhibition and supports further evaluation of nitroalkenes as therapeutic agents in RECQL4-amplified cancers.

## INTRODUCTION

Nitroalkenes are a class of electrophilic lipids endogenously produced by the non-enzymatic nitration of dietary unsaturated fatty acids (e.g., conjugated linoleic acid) by nitric oxide and other nitrite-derived free radical species^1–4^. Nitroalkenes are electrophiles that reversibly modify nucleophilic protein cysteine thiols through the formation of a covalent bond between the electron-poor β-carbon adjacent to the nitroalkene double bond via a Michael addition reaction, and the sulfur atom of cysteine residues that have been reduced to their thiolate anion form^5, 6^. Nitroalkenes are produced primarily during gastric acidification and under inflammatory stress conditions and are detectable in human plasma and urine at low nanomolar concentrations^4, 7, 8^. It is widely recognized that nitroalkenes and other electrophilic lipid species function as signaling molecules and mediate many anti-inflammatory health benefits of dietary nitrite and omega-3 polyunsaturated fatty acids^2, 7^. The anti-inflammatory effects of nitroalkenes vary and are driven by the alkylation of multiple important protein targets (see^1^ for a comprehensive review). Previous reports demonstrated that micromolar concentration doses of exogenous nitroalkenes elicit similar responses as long-term exposure to endogenous physiological nitroalkene concentrations^1, 5^. Thus, nitroalkenes have been extensively evaluated as pharmacological agents with considerable success: CXA-10, a derivative of the nitroalkene 10-nitro-oleic acid (OA-NO_2_), has been tested in multiple Phase II clinical trials against chronic renal and cardiopulmonary disorders and exhibits excellent tolerability at therapeutically effective doses (300 mg/day, PO)^9, 10^.

Surprisingly, in contrast to its canonical cytoprotective role in inflammatory disease models, OA-NO_2_ exhibits single-agent toxicity in triple-negative breast cancer (TNBC) *in vitro* and *in vivo* and sensitizes TNBC to multiple clinically relevant antineoplastic DNA damaging agents, including ionizing radiation (IR), cisplatin, and Poly-ADP Ribose Polymerase (PARP) inhibitors^11,12^. Recently, we generated a novel small molecule nitroalkene library that facilitated the discovery of a compound, designated CP-8, that was superior to OA-NO_2_ both in its ability to suppress TNBC growth and in its ability to sensitize TNBC cells to other DNA-damaging therapies^13^. The synergistic effects of nitroalkenes and DNA-damaging agents combined with their cytoprotective effects in benign tissue^12, 13^ motivate interest in exploring nitroalkenes as a novel class of safe and tolerable anti-cancer therapeutics.

Insights into the mechanism of nitroalkene-mediated cancer suppression indicate that the anti-cancer effects of nitroalkenes are multifaceted. Nitroalkene-mediated suppression of constitutive pro-inflammatory Nuclear Factor kB (NF-kB) signaling is required for the nitroalkene-mediated prevention of TNBC growth and metastasis *in vitro*^12^. Complementary work using colorectal cancer as a model indicated that OA-NO_2_ treatment also induces mitochondrial oxidative stress and activates caspase-dependent apoptosis both *in vitro* and in xenograft mouse models^14^. Further mechanistic work demonstrated that OA-NO_2_ alkylates the essential recombinase RAD51 at Cys319, preventing RAD51 nucleoprotein filament formation and suppressing homologous recombination (HR)-dependent DNA double-strand break (DSB) repair^11^. However, given their multi-target effects, no single identified mechanism can fully account for the variety of nitroalkene anti-cancer activities^12, 13^.

To that end, TMT-labeling coupled with liquid chromatography and tandem mass spectrometry (LC-MS/MS) was used to characterize the nitroalkene cancer proteome better using U2OS osteosarcoma cells as a model system. This analysis identified the multifunctional helicase RECQL4 as a novel nitroalkene target and determined the modified amino acid residue to be Cys1052. This interaction was confirmed by chemoproteomics and revealed to be DNA-damage dependent. While Cys1052 has not yet been identified as a functionally significant residue, structural analysis of RECQL4 revealed that Cys1052 resides in a functionally significant domain of RECQL4 and exhibits characteristics compatible with nucleophilicity. Biochemical and cell-based analyses revealed that OA-NO_2_ inhibits RECQL4 helicase activity, disrupts DSB end resection, and blocks downstream homology-dependent DSB repair. Furthermore, mutation of Cys1052 to a serine phenocopies the effects of OA-NO_2_ on RECQL4 recruitment to DSBs and rescues the effects of OA-NO_2_ on DSB end resection. Finally, overexpression of WT RECQL4 but not C1052S mutant protein was sufficient to blunt the impact of OA-NO_2_ on DSB repair and cell viability, supporting RECQL4 as a key mediator of nitroalkene-dependent DSB repair inhibition.

## RESULTS

### LC-MS/MS reveals redox-dependent regulation of the cancer proteome by OA-NO_2_

**Figure 1:**
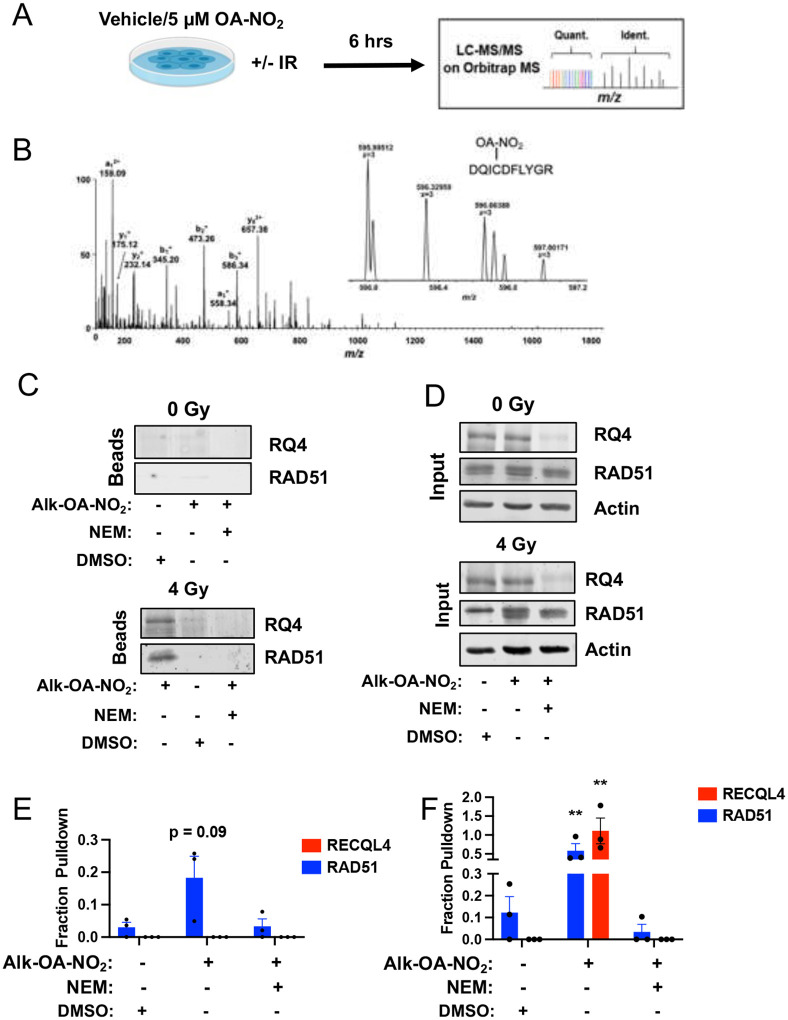
Chemoproteomics reveals RECQL4 Cys1052 as a novel nitroalkene target in cancer. (**A**) Schematic depicting the design of TMT-labeled LC-MS/MS analysis to identify novel nitroalkene targets in the cancer proteome. (**B**) Peptide ion spectrum of RECQL4 with the fragment containing Cys1052 mass shifted by the molecular weight of OA-NO_2_ indicated by a red arrow. (**C and D**) Representative blot of CuAAC enrichment of RECQL4 and RAD51 by Alk-OA-NO_2_ in the presence of 0 and 4 Gy IR. Note the different sample arrangements in D compared to C. (**D**) Fold enrichment of RECQL4 and RAD51 relative to input in the presence of IR. Mean +/− SEM of N=3 independent experiments. Statistical significance was calculated by two-way ANOVA followed by Tukey’s test for multiple comparisons. **, p < 0.01 (**E**) Representative blot of CuAAC enrichment of RECQL4 and RAD51 in cells treated with 0 Gy IR. (**F**) Fold enrichment RECQL4 and RAD51 relative to input in cells treated with 4 Gy IR. Mean +/− SEM of N=3 independent experiments. Statistical significance was calculated by two-way ANOVA followed by Tukey’s test for multiple comparisons.

Nitroalkenes are promising tumor-selective antineoplastic agents with a strong safety profile shown in preclinical models and clinical Phase 1 and 2 trials^15^. To characterize the multifactorial effects of nitroalkenes, proteins were extracted from U2OS osteosarcoma cells 6 hours after OA-NO_2_ treatment, then digested and labeled with unique tandem mass tags (TMT) for quantification via liquid chromatography coupled to tandem mass spectrometry (LC-MS/MS). The goals were to evaluate the global effects of nitro-alkylation on cancer signaling and identify novel nitroalkene targets (Fig. 1A). Given a recent published study showing OA-NO_2_ inhibiting homologues recombination (HR) mediated DNA repair^11^, U2OS cells were chosen due to their well-characterized DNA repair systems, ideal for pinpointing potential targets. The experiment was conducted with or without IR to initiate DNA damage and assess how nitroalkene-regulated signaling responds to redox changes, given their nucleophilicity^16, 17^.

LC-MS/MS analysis identified around 439,000 peptides corresponding to about 8,392 unique proteins. Unsupervised clustering indicated that treatment conditions were better defined by drug treatment than IR exposure, with OA-NO_2_ treated samples more closely related than controls. Most proteins altered after OA-NO_2_ treatment were downregulated relative to controls (Extended Data Fig. 1A). Notably, over 80% of differentially regulated proteins were unique to the presence or absence of IR (Extended Data Fig. 1B). This supports the idea that redox changes influence the cysteine proteome and thiol susceptibility to nitroalkenes^18, 19^.

To understand nitro-alkylation impacts on signaling, proteins with an absolute log2 fold change > 2 after OA-NO_2_ treatment in both irradiated and non-irradiated cells underwent gene ontology (GO) pathway analysis to identify the top 5 biological processes affected. Among the top 20 GO terms impacted by OA-NO2 across IR conditions, 25% related to mitochondrial processes, including RNA processing and mitophagy (Extended Data Fig. 1C–D). This aligns with findings that the mitochondrial niche is rich in nitroalkene production and signaling^20, 21^. Additionally, 40% (4/10) of GO biological process terms downregulated by OA-NO_2_ were related to cell growth and division (Extended Data Fig. 1C–D). Remarkably, with IR, 60% of downregulated terms involved fatty acid metabolism (Extended Data Fig. 1C), implying OA-NO_2_ may induce an adaptive loop to shield the proteome from electrophilic modifications. The findings suggest that nitro-alkylation effects on cancer signaling are adaptive and vulnerable to redox changes.

### OA-NO_2_ alkylates RECQL4 at C1052

A secondary endpoint of the LC-MS/MS analysis was to detect the alkylation of novel protein targets by OA-NO_2_ by identifying peptides mass-shifted by the approximate mass of OA-NO_2_. This analysis revealed multiple peptides from 20 unique proteins modified by OA-NO_2_ (Table 1). Notably, from a DNA repair perspective, the versatile ATP-dependent helicase-like RECQL4 was alkylated at Cys1052 (Table 1, Fig. 1B). RECQL4, a conserved member of the 3’-5’ RecQ helicase family, is crucial in DNA replication and repair^22–25^. Viable mutations in RECQL4 that impair helicase activity led to three autosomal-recessive disorders: Rothmund-Thompson, RAPADLINO, and Baller-Gerold Syndromes, which involve premature aging and cancer predisposition^22, 26^.

To confirm the OA-NO_2_ and RECQL4 interaction at Cys1052, we employed a copper-assisted azide-alkyne cycloaddition (CuAAC) chemoproteomics approach to enrich nitroalkene targets. CuAAC is a variety of click chemistry^27–29^. CuAAC utilizes copper (I) ions for rapid and efficient bio-orthogonal labeling of proteins under physiological conditions, catalyzing a Huisgen-1,3-dipolar cycloaddition reaction^30^. Following established CuAAC methods^31–33^, U2OS cells were labeled with Alk-OA-NO_2_. CuAAC conjugated Alk-OA-NO_2_ proteins to a diazo biotin azide tag upon cell lysis. Biotinylated complexes were isolated via streptavidin pull-down, eluted, and analyzed using electrophoresis and western blot (Extended Data Fig. 2A). The CuAAC protocol labeled U2OS lysates dose-dependently with biotin and enriched for the validated nitroalkene targets β-actin and RAD51 (Extended Data Fig. 2B).

In the absence of IR, Alk-OA-NO_2_ weakly enriched RAD51 proteins (18% enrichment, p = 0.09) and did not appear to interact with RECQL4 (Fig. 1E–F). In contrast, under IR, Alk-OA-NO_2_ treatment significantly enriched for both RECQL4 (98% enrichment, p < 0.01) and RAD51 (60% enrichment, p < 0.01) (Fig. 1C–D). Pre-saturation of thiols with N-ethylmaleimide abolished enrichment (Fig. 1C–F). This indicates that the interaction between OA-NO_2_ and RECQL4 relies on conditions present during DNA damage that affect Cys1052’s nucleophilicity.

### RECQL4 Cys1052 resides in a functionally significant domain

**Figure 2:**
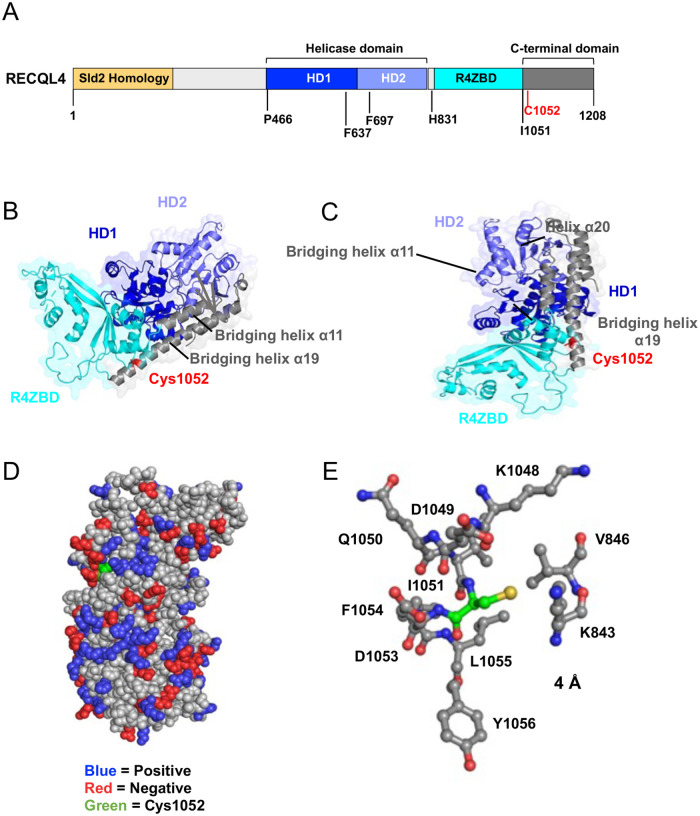
Structural analysis of RECQL4 Cys1052 reveals possible functional role. (**A**) Domain diagram of full-length RECQL4 with Cys1052 highlighted in red. Mutations denoted in black have previously been identified to inhibit RECQL4 helicase activity in humans. (**B and C**) Structure of RECQL4^449–1111^ in ribbon presentation in two views with the two RecA-like domains HD1 and HD2 shown in lighter and darker blue, the R4ZBD domain in cyan, and the C-terminal domain in grey, respectively. Cys1052 is highlighted in red. (**D**) Space-filling model of RECQL4^449–1111^ with Cys1052 (green) and positively (blue) and negatively (red) charged residues highlighted. (**E**) Ball-and-stick model depicting all amino acid residues located within a 4 Å radius of Cys1052 (thiol denoted in yellow). (**A**) Domain diagram of full-length RECQL4 with Cys1052 highlighted in red. Mutations denoted in black have previously been identified to inhibit RECQL4 helicase activity in humans. (**B and C**) Structure of RECQL4^449–1111^ in ribbon presentation in two views with the two RecA-like domains HD1 and HD2 shown in lighter and darker blue, the R4ZBD domain in cyan, and the C-terminal domain in grey, respectively. Cys1052 is highlighted in red. (**D**) Space-filling model of RECQL4^449–1111^ with Cys1052 (green) and positively (blue) and negatively (red) charged residues highlighted. (**E**) Ball-and-stick model depicting all amino acid residues located within a 4 Å radius of Cys1052 (thiol denoted in yellow).

Structural analyses of RECQL4 Cys1052 were conducted to understand its functional properties. Unlike most disease-causing mutations affecting RECQL4 helicase function, Cys1052 is in the C-terminal domain, not near the helicase or zinc-binding domains^34^ (Fig. 2A). The main feature of this domain is a bundle of three α-helices: α11 (823–835aa), α19 (1046–1079aa), and α20 (1094–1109aa), where α11 and α19 connect the helicase domains^35^ (Fig. 2B–C). Cys1052 is at the N-terminal region of helix α19, next to Ile1051, a residue mutated to valine in a Rothmund-Thompson Syndrome patient^26^ adjacent to an alpha-helical motif (Fig. 2A–C).

Cys1052 is solvent-exposed, positioned between two oppositely charged amino acid clusters (Fig. 2D). Within 4 Å of Cys1052, it is next to Ile1051 and several charged residues (Fig. 2E). Being close to positively charged residues raises the likelihood of nearby cysteines being reduced to thiolate anions and susceptible to electrophilic modification^36^. Our analysis suggests Cys1052 is nucleophilic and functionally relevant. Thus, we evaluated the effects of nitro-alkylation on RECQL4 helicase activity and downstream DSB repair using biochemical and cell-based assays.

### OA-NO_2_ disrupts the ATP-dependent RECQL4 biochemical helicase activity

**Figure 3:**
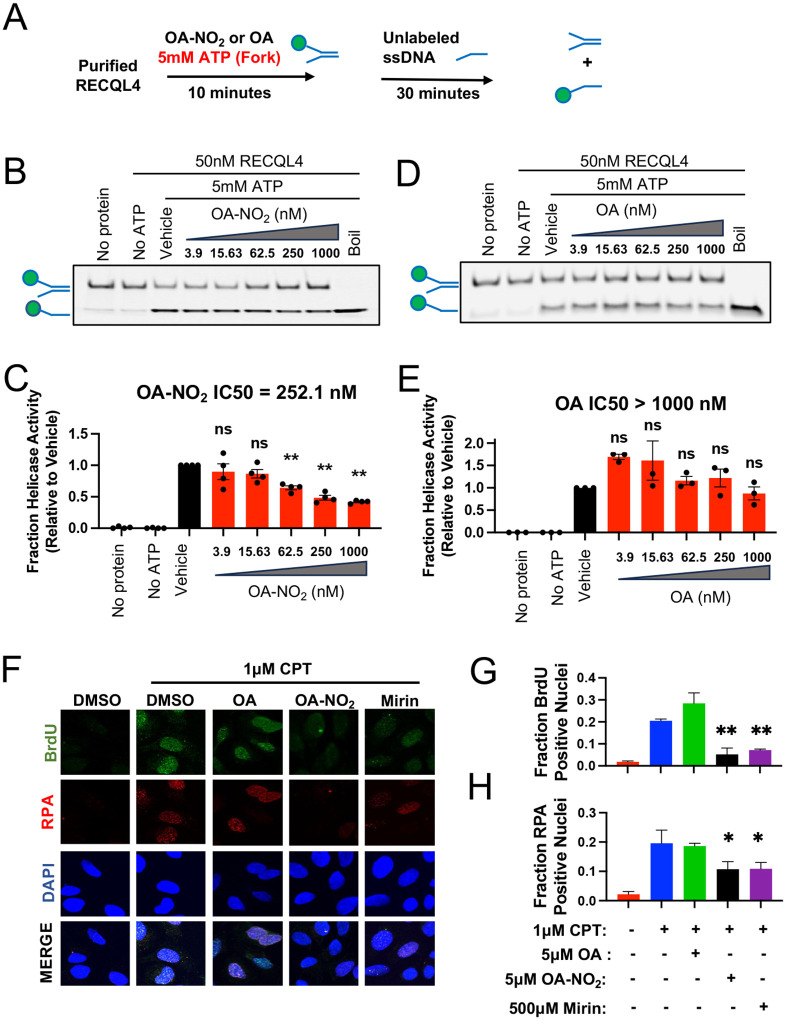
OA-NO_2_ disrupts RECQL4 helicase activity, inhibiting DSB end resection and downstream homology-dependent DSB repair in cells. (**A**) Schematic depicting the design of the helicase assay. (**B and D**) Representative blots of the dose-dependent effect of OA-NO_2_ and OA on RECQL4 helicase-dependent ssDNA production. Relative positions of the double and single-stranded substrate are labeled on each gel. (**C and E**) Helicase activity was quantified as ssDNA production relative to the vehicle. Mean +/− SEM of N=3 independent experiments. IC_50_ values were calculated by fitting non-linear regression curves of helicase activity data. Statistical significance was calculated by one-way ANOVA followed by Tukey’s test for multiple comparisons. ns, not significant. **, p < 0.01. (**F**) Representative images of the effect of OA-NO_2_, OA, and Mirin on CPT-induced ssDNA formation in RECQL4-proficient U2OS cells. (**G and H**) Quantification of ssDNA formation as measured by the fraction of BrdU or RPA positive nuclei, respectively. Data was plotted as mean + SEM of N=3 independent experiments. Statistical significance was calculated by one-way ANOVA followed by multiple comparison testing. *, p < 0.05. **, p < 0.01.

To assess the significance of nitro-alkylation of RECQL4, purified RECQL4 without its N-terminus (RECQL4^449–1111^) was treated with OA-NO_2_ and ATP, evaluating unwinding of a fluorescently labeled 30nt forked oligonucleotide duplex (Fig. 3A). In the absence of purified protein or ATP, most labeled oligo remained double-stranded. However, with ATP, RECQL4^449–1111^ generated fluorescently labeled single-stranded oligo, inhibited by OA-NO_2_ (IC50 = 252.1nM), reaching maximum inhibition of ~50% compared to vehicle at the highest dose tested (p < 0.01) (Fig. 3B–C). Non-specific fatty acid interactions were ruled out, as pre-treatment with oleic acid (OA) did not inhibit RECQL4^449–1111^ helicase activity (IC50 > 1000nM) (Fig. 3D–E) and trended towards increased helicase activity, though not statistically significant.

Using a real-time fluorescence assay, we also validated OA-NO_2_’s inhibitory effect on RECQL4 helicase, confirming dose-dependent inhibition on unwinding a short 19nt oligo duplex^35^ (Extended Data Fig 3A). Similar to the gel-based assay results, OA-NO2 inhibited helicase activity by ~40% at the highest dose tested (p < 0.01) (Extended Data Fig. 3B–C), establishing OA-NO_2_ as the first small molecule inhibitor of RECQL4 helicase activity. Since RECQL4 helicase requires ATPase activity, we tested nitroalkene-dependent inhibition’s reliance on ATP binding in the gel-based assay without ATP during pre-treatment (Extended Data Fig. 3D). Following prior data, 1μM OA-NO_2_ with ATP decreased helicase activity by ~50% (p < 0.01) compared to DMSO control. In contrast, pre-treatment without ATP showed no effect (Extended Data Fig. 3E–F), suggesting nitro-alkylation of RECQL4 likely depends on ATP binding.

### OA-NO_2_ prevents DSB end resection and inhibits downstream homology-dependent DSB repair.

We examined if OA-NO_2_ disturbs RECQL4-dependent DSB repair, focusing on DSB end resection. OA-NO_2_ was evaluated for its impact on ssDNA formation and RPA accumulation in U2OS cells treated with the topoisomerase II inhibitor camptothecin (CPT) to induce DSBs^37, 38^. U2OS cells were labeled with the thymidine analog BrdU and treated with CPT with or without pre-treatment with OA-NO_2_. Nuclei were subsequently isolated and stained with antibodies against BrdU and the DNA binding protein Replication Protein A (RPA) to quantify ssDNA formation by immunofluorescence.

CPT alone significantly increased nuclear ssDNA as quantified by BrdU and RPA (Fig. 3F–H). Mirin, an MRE11 exonuclease inhibitor, reduced ssDNA formation (~10% for BrdU, p < 0.01; ~15% for RPA, p < 0.05). At a much lower dose, OA-NO_2_ mimicked mirin’s effect with a ~15% reduction in both BrdU and RPA positive nuclei. However, OA pre-treatment did not rescue ssDNA formation (BrdU: ~8% increase, ns; RPA: ~6% reduction, ns) (Fig 3H–H). This effect wasn’t due to OA-NO_2_’s modification of the cell cycle or inhibition of total BrdU accumulation in DNA (Extended Data Fig. 4A–C).

We have recently shown that OA-NO_2_ inhibits HR but does not inhibit the homology-independent repair pathway classical non-homologous end joining (C-NHEJ)^11^, prompting investigation of its effects on other DSB repair mechanisms like single-strand annealing (SSA) and alternative end-joining (Alt-EJ). U2OS GFP reporter cell lines^39, 40^ were transfected with the restriction enzyme I-SceI to induce DSBs, treated with OA-NO_2_ for 48 h, followed by GFP expression analysis via flow cytometry. OA-NO_2_ significantly inhibited SSA (0.15% reduction, p < 0.05) and Alt-EJ (1% reduction, p < 0.01) (Extended Data Fig. 4D–I), indicating its influence on DSB repair upstream of SSA, Alt-EJ, and HR at the level of end resection by modulating RECQL4 activity through adducting Cys1052.

### RECQL4 C1052S decreases RECQL4 recruitment to DSBs and phenocopies the effect of OA-NO_2_ on DSB end resection

To explore Cys1052’s role in end resection and its mimicry of OA-NO_2_ effects on RECQL4, a Cys1052Ser mutant was compared to OA-NO_2_ treatment for changes in recruitment or retention of GFP-tagged RECQL4 to laser-generated DNA DSB sites.

**Figure 4:**
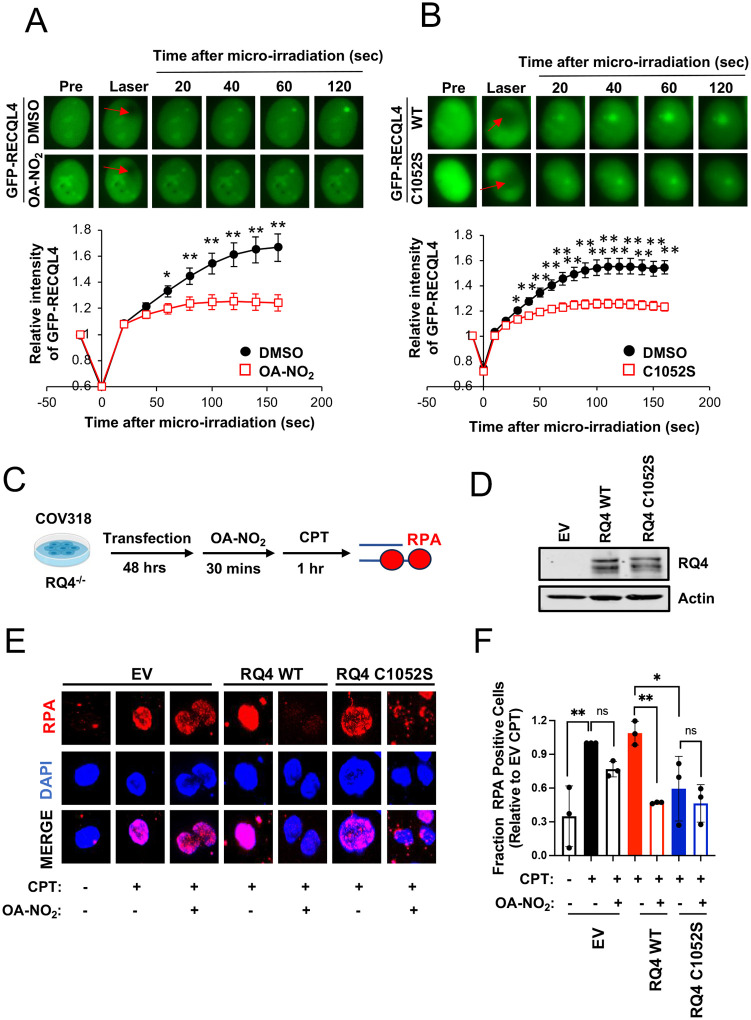
The RECQL4 C1052S variant phenocopies nitro-alkylation of RECQL4 and rescues the effect of OA-NO_2_ on DSB end resection. (**A**) The effect of OA-NO_2_ on recruitment of GFP-RECQL4 to sites of laser-induced micro-irradiation over time. Red arrows indicate sites of irradiation. Recruitment was quantified as the relative GFP intensity at the irradiation site over time. Mean +/− SEM of N = 9–17 independent micro-irradiation events. The students’ t-tests quantified statistical significance at each time point tested. *, p < 0.05. **, p < 0.01. (**B**) Recruitment of WT GFP-RECQL4 and GFP-RECQL4-C1052S to sites of laser-induced micro-irradiation over time. Red arrows indicate sites of irradiation. Mean +/− SEM of N = 22–26 independent micro-irradiation events. Statistical significance was calculated using the student’s t-tests at each time point tested. *, p < 0.05, **, p < 0.01. (**C**) Schematic depicting the design of an immunofluorescence experiment to test the effect of OA-NO_2_ treatment on end resection in RECQL4^−/−^ COV318 cells by measuring ssDNA formation in intact nuclei. (**D**) Representative reconstitution blot of RECQL4 with either WT or the C1052S variant in RECQL4^−/−^ COV318 cells. (**E**) Representative IF images of the effect of reconstitution with WT or the C1052S variant RECQL4 on the ability of OA-NO_2_ to suppress CPT-induced ssDNA formation in COV318 cells. (**F**) ssDNA formation was quantified as the fraction of RPA-positive cells relative to CPT-treated cells in the absence of RECQL4 reconstitution. Mean +/− SEM of N = 3 independent experiments with at least 100 nuclei measured in each treatment condition. Statistical significance was quantified by two-way ANOVA followed by Tukey’s test for multiple comparisons. ns, not significant. *, p < 0.05. **, p < 0.005.

In DMSO-treated cells, GFP-RECQL4 quickly recruited to laser-induced micro-irradiation, showing a 60% intensity increase at 120 s post-irradiation (Fig. 4A). Conversely, OA-NO_2_ pre-treatment significantly reduced GFP-RECQL4 intensity by 21% at 60 s post-irradiation (p < 0.05) and caused a maximum decrease of ~60% at the last timepoint (p < 0.01) (Fig. 4A). The recruitment kinetics of GFP-tagged RECQL4 were similar to WT protein treated with OA-NO2, both showing a maximum intensity reduction of ~60% (p < 0.01) (Fig. 4B). This data indicates OA-NO_2_ pre-treatment markedly inhibits RECQL4 recruitment to laser-induced DSBs and that mutation of Cys1052 to a serine phenocopies this effect. This supports the hypothesis that Cys1052 is the residue that mediates nitroalkene-dependent disruption of RECQL4 recruitment to DSBs.

To investigate whether RECQL4 specifically mediates OA-NO_2_’s effect on DSB end resection, the impact of OA-NO_2_ on ssDNA production was evaluated in RECQL4−/− ovarian cancer cells reconstituted with WT or mutant RECQL4 (Fig. 4C–D). ssDNA formation was quantified using RPA intensity due to the slow doubling time of these cells limiting BrdU incorporation (data not shown). In these cells, CPT treatment induced a 65% increase in RPA intensity, while OA-NO_2_ pre-treatment led to a 20% decrease in RPA intensity without statistical significance (Fig. 4E–F). However, reconstituted WT RECQL4 significantly enhanced OA-NO_2_’s inhibition of CPT-induced ssDNA formation (~60% reduction in RPA intensity, p < 0.005) (Fig. 4E–F). Meanwhile, reconstituted RECQL4 mutants decreased CPT-induced ssDNA formation by ~50% compared to CPT in EV cells (p < 0.05), aligning with evidence that RECQL4 mutant expression mimics nitro-alkylation effects. Additionally, the effect of OA-NO_2_ on preventing CPT-induced ssDNA in RECQL4 mutants was similar to that in EV control cells (~30% decrease compared to CPT alone, ns) (Fig. 4E–F). These findings indicate that RECQL4 Cys1052 is necessary for OA-NO_2_’s suppression of DSB end resection, confirming RECQL4 as the primary mediator in this process.

### High RECQL4 levels in cancer correlate with genomic instability

**Figure 5:**
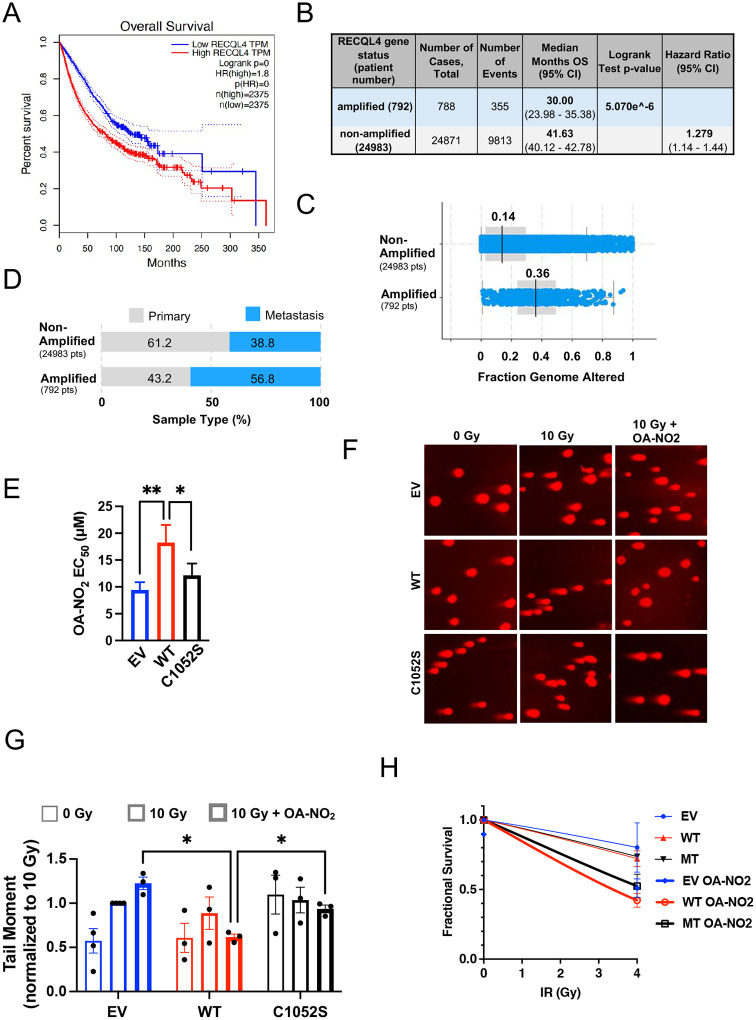
High RECQL4 levels correlate with poor patient survival in a pan-cancer analysis and are targeted by OA-NO_2_ in vitro (**A**) Overall survival (OS) plot depicting upper (cutoff-high quartile 75%) and lower (cutoff low quartile 25%) RECQL4 mRNA expression patient groups estimated by Mantel-Cox analysis. Abbreviations describing cancer types are available in Extended Data Table 1. (**B**) The MSK Met Tropism cohort in cbioportal was analyzed for OS, which depends on RECQL4 amplification from a pan-cancer cohort^43–46^. (**C**) Fraction Genome Altered (FGA) analysis from cohort in B. The FGA is calculated by dividing the number of genomic regions with significant copy number changes by the total size of the genome analyzed. Chi-squared statistical test analysis, p<10^-10, q<10^-10. (**D**) Metastases analysis from patient cohort described in B. Chi-squared statistical test analysis, p<10^-10, q<10^-10. (**E**) U2OS OA-NO_2_ EC_50_ values were calculated in cells irradiated and treated with OA-NO_2_ (increasing concentrations from 0 to 20 μM) just before irradiation (10 Gy) by fitting cell viability data to a non-linear variable slope model. Mean +/− SEM of N=3–4 independent experiments. Significance was calculated using two-way ANOVA followed by multiple comparisons testing. *, p < 0.05 and **, p < 0.005. (**F-G**) Images of a neutral comet assay of U2OS cells treated with IR and OA-NO_2_. Mean +/− SEM of N=3–4 independent experiments. (**H**) U2OS clonogenic survival assay of cells treated with 10 μM OA-NO_2_ and IR (0 and 4 Gy) once 24h after cell plating. Colonies were counted and analyzed using ImageJ quantification. Mean +/− SEM of N=2 independent experiments. Plot analysis was done in GraphPad using the linear quadratic equation Y=AX+BX^2.

We used three interactive web servers to examine the correlation between RECQL4 expression and cancer patient overall survival (OS). The GePIA pan-cancer database (gepia.cancer-pku.cn) utilized RNA-seq expression data from 9,736 tumors and 8,587 normal samples. High RECQL4 transcript per million (TPM) levels in 31 tumor types correlated significantly with shorter patient OS (HR of 1.8) (Fig. 5A). Of these, 28 had higher RECQL4 TPMs compared to normal tissue (Extended Data Fig. 5A). To assess if high mRNA RECQL4 expression relates to cancer genomic instability, we utilized pan-cancer data from the cBioPortal that examined copy number alterations and mutations. Among 12 available pan-cancer sets, only ICGC/TCGA provided RECQL4 mRNA data with copy number alterations. In 14% of samples (2,922 samples / 2,583 patients), high mRNA or gene amplifications correlated with lower patient OS (HR of 2.172, 95% CI 1.172 – 4.017; log-rank p-value: 9.085e-4). Patients with increased RECQL4 mRNA or gene amplifications showed a higher frequency of metastasis and genomic instability (Extended Data Fig. 5B). Given the limited patient group in this data set, a larger dataset with 25775 samples was analyzed for RECQL4 mutations, structural variants, and copy number alterations. As shown in Fig. 5B, RECQL4 gene amplifications were found in 792 of the 25775 samples, significantly correlating with decreased OS (HR: 1.279, log-rank test p value: 5.07e^-6), increased genomic instability (Fraction of Genome Altered, FGA) (Fig. 5C), and metastasis count per patient (Fig. 5D). RECQL4 copy number alterations correlated with specific cancer types, including prostate, ovarian, non-small cell lung, melanoma, colorectal, and breast cancer (Extended Data Fig. 5C). Analyzing survival by cancer type using the KmPlotter Server showed that increased RECQL4 levels correlated with shorter survival in ovarian, breast, lung, and pancreatic cancer (Extended Data Fig. 5D–G). These findings indicate that RECQL4 overexpression strongly correlates with metastasis, genomic instability, and shorter patient survival and highlights the potential survival benefits of targeting RECQL4 in various cancers.

### Overexpression of WT but not RECQL4 C1052S mutant protein partially rescues the effects of OA-NO_2_ on DSB repair and cell viability.

Next, we investigated the contribution of RECQL4 inhibition by OA-NO_2_ to DNA damage. U2OS and Cov318 cells were transfected with plasmids for the WT or RECQL4 C1052S mutant protein. These cells were dosed with OA-NO_2_ before exposure to IR or CPT and then DSB formation was grossly assessed by γ-H2AX phosphorylation. Given our previous studies demonstrating the HR-essential recombinase RAD51 as a nitroalkene target^11, 13^, EV cells showed increased γ-H2AX phosphorylation 3 h after IR or CPT, which OA-NO_2_ exacerbated.

### Overexpression of WT but not RECQL4 C1052S mutant protein partially rescues the effects of OA-NO_2_ on DSB repair and cell viability.

Next, we investigated the contribution of RECQL4 inhibition by OA-NO_2_ to DNA damage and cell survival. To establish potential changes in OA-NO_2_ EC50 values by the overexpression of RECQL4, U2OS and Cov318 cells were transfected with plasmids for the WT or RECQL4 C1052S mutant protein, treated with increasing concentrations of OA-NO_2_ before IR treatment. After 24 h, cells were stained with crystal violet to measure cell survival. Irradiated RECQL4 WT-OE cells showed an almost 2-fold higher OA-NO_2_ EC50 than EV cells (18.25μM and 9.4μM, respectively) and a 1.5-fold higher EC50 compared to C1052S expressing cells (18.25μM and 9.4μM, respectively), further confirming RECQL4 as an OA-NO_2_ target (Fig. 5E). To assess then DNA DSB more specifically, U2OS cells expressing RECQL4 WT and the C1052S variant were analyzed by neutral comet assay. Cells were exposed to EV cell EC50 doses of OA-NO_2_ (10 μM) before irradiation (10 Gy) and examined after 3 h. Mirroring the increase in OA-NO_2_ EC50, IR treatment increased DNA DSB formation (Tail moment) in EV cells. In contrast, however, OA-NO_2_ treatment lowered DNA DSB formation in irradiated RECQL4 WT-OE cells compared to EV and RECQL4 C1052S-OE cells (Fig. 5F–G). Similarly, γ-H2AX phosphorylation was exacerbated in EV cells treated either with IR or CPT, which OA-NO_2_ could reduce in RECQL4 WT-OE Cov318 and U2OS cells (Extended Data Fig. 5 H–J). As the timing of our DNA DSB formation assays did not account for any long-term effects of RECQL4 inhibition by OA-NO_2_, we explored U2OS cells treated with the EV EC50nof OA-NO_2_ 10 μM before 4 Gy irradiation. As shown in Fig. 5G, OA-NO_2_ treatment in RECQL4 WT OE cells decreased colony formation in irradiated cells more than in EV and C052S OE cells, supporting the value of targeting RECQL4 with OA-NO_2_ in IR-treated cells.

## DISCUSSION

Our results demonstrate that RECQL4 is a novel target for nitroalkene-mediated inhibition of its helicase activity by nitroalkylating the C-terminal RECQL4 cysteine C1052. Nitroalkenes modify protein cysteines through the Michael Addition reaction and are promising multi-target anti-cancer drugs^15^. However, their mechanism of action is not fully understood. Here, we present further mechanistic evidence that the pleiotropic anti-cancer properties of nitroalkenes^15^ arise partly from their impact on DNA double-strand break (DSB) repair by inhibiting the helicase activity of RECQL4 through the nitro-alkylation of Cys1052.

The global proteomics analysis revealed that IR exposure alters protein modification by OA-NO_2_ in cancer cells. IR exposure shifted the proteins modified by OA-NO_2_ in the cancer proteome. IR induce reactive oxygen species (ROS)^48^. As tumors already generate higher levels of ROS due to hypoxia and dysregulated redox homeostasis driven by oncogenes^49^, this heightened oxidative stress probably renders a subset of the cysteine proteome more vulnerable to electrophilic modification. The evidence presented here indicates that this redox perturbation affects the cysteinyl proteome sensitive to nitro-alkylation: non-irradiated cells showed significantly less OA-NO_2_ modification of RECQL4 and RAD51, known targets of nitroalkenes. Important to note here is that IR also induces other regulatory PTMs on RECQL4, such as phosphorylation by DNA-PKs following IR-treatment^50^, that may also contribute to conformational changes in RECQL4 that expose Cys1052 to OA-NO_2_ adduction.

LC-MS/MS analysis of nitro-alkylation in U2OS cells identified 20 novel nitroalkene targets, pinpointing specific cysteines alkylated by OA-NO_2_. A similar study of 9-nitro-octadec-9-enoic acid (9-NO_2_-FA) in Caco2 colorectal cancer cells identified around 30 novel targets^51^. It is thus plausible that the low number of resolvable targets may be an intrinsic limitation of this type of analysis and may stem from difficulties in distinguishing a few alkylated cysteinyl peptides among thousands in global proteomics. Future efforts to discover nitroalkene targets in other cell models may need to first concentrate nitroalkene targets, possibly through CuAAC-based methods, before peptide identification.

A combination of biochemical and cell-based assays revealed that nitro-alkylation of RECQL4 by OA-NO_2_ disrupts helicase activity and inhibits DSB end resection. One intriguing observation from these experiments was that ATP was necessary for nitro-alkylation of RECQL4. This has important implications for how nitro-alkylation may affect RECQL4 biology because the necessity of ATP for RECQL4 helicase function largely depends on the context in which RECQL4 is required. For example, the role of RECQL4 in DNA replication depends entirely on its unstructured N-terminus, which demonstrates significant strand annealing activity in biochemical assays^52–54^, and depletion of the entire C-terminus of RECQL4 does not perturb DNA replication in human B-cell progenitors *in vitro*^55^.

The C-terminus and its associated ATPase activity are necessary for DSB repair in human cells^24, 55–57^. However, reports in mice that a RECQL4 K525A mutant lacking ATP binding did not affect DSB repair^58^. Thus, tools that selectively separate the C-terminal and N-terminal functionalities of RECQL4 are required to understand these conflicting reports. The biochemical assays discussed here used recombinant RECQL4, lacking the N-terminus^35^. Thus, it was impossible to assess the viability of OA-NO_2_ as a separation-of-function agent in vitro. Future biochemical experiments with full-length RECQL4 can test if OA-NO_2_ affects strand annealing activity. However, this may prove challenging as full-length RECQL4 is notoriously difficult to express and purify^35^.

Our data supports a mechanistic model of nitro-alkylation of RECQL4, where it mainly exists in its unbound ATP state, keeping Cys1052 thiol protonated and thus insensitive to nitro-alkylation. This explains the negligible interaction between RECQL4 and OA-NO_2_ without DNA damage. After DNA damage and ATP binding, changes in RECQL4 promote formation of the Cys1052 thiolate anion, increasing sensitivity to nitro-alkylation and inhibiting end resection and homology-dependent DSB repair. Currently, this model lacks mechanistic detail on how ATP binding affects Cys1052 nucleophilicity, which can be addressed in future studies examining crystal structures of RECQL4 with ATP and DNA. Structural evidence suggests the C-terminus may stabilize helicase orientation^35^, leading to speculation that nitro-alkylation could destabilize RECQL4’s proper orientation and affect helicase function. Supporting this, RECQL4 C1052S has lower expression than RECQL4 WT in COV318 cells (data not shown). However, confirming this requires direct evidence that OA-NO_2_ destabilizes RECQL4.

Experiments with the RECQL4 C1052S mutant protein show that Cys1052 is essential for its nitro-alkylation and is a key functional residue in RECQL4 biology. This finding is significant, as identifying covalent inhibitors for disease-relevant cysteines aids in designing inhibitors for challenging cancer targets^59^. For instance, the oncogenic kinase KRAS was deemed undruggable until covalent electrophiles for mutant KRAS G12C emerged^60^. Covalent cysteine-targeting inhibitors have also been developed for other RecQ helicases. VVD-133214 is a newly discovered small molecule that allosterically modifies WRN Cys727, hindering necessary interdomain interactions for WRN helicase activity and causing synthetic lethality in MSI-high colorectal cancer cells^61^. Likewise, screening small molecule electrophile probes against the SF1-family of SARS-CoV-2 helicases nsp13 revealed inhibition of other mammalian helicases, including BLM and WRN^62^.

Loss of RECQL4 helicase activity predisposes patients to premature aging and cancers in Rothmund-Thompson, RAPADLINO, and Baller-Gerold Syndromes^26^. In breast cancer, RECQL4 prevents abnormal recombination during DNA repair of cisplatin-induced DNA cross-links^22^. Higher RECQL4 levels correlate with poor prognosis in cancer of the breast, liver, stomach, prostate, ovarian, and skin (melanoma)^22, 63–68^. RECQL4 depletion in oxaliplatin-resistant colon adenocarcinoma and prostate cancer decreased oncogenic behaviors. Our pan-cancer database screen supports that high RECQL4 levels are associated with genomic instability, metastasis, and poor outcomes in patients with elevated RECQL4 mRNA in tumors and with RECQL4 gene amplification. While the detailed mechanisms by which high RECQL4 promotes genomic instability remain unclear, similar outcomes have been noted for other DNA repair proteins, including RAD51^69^, that RECQL4 and other RecQ helicases have been focused on in recent drug development efforts^13, 70, 71^.

The results presented here suggest that OA-NO_2_ is a novel inhibitor of RECQL4 helicase activity. Treatment with OA-NO_2_ significantly reduced RECQL4 helicase activity and ssDNA recruitment in endresection in vitro, which the C1052S RECQL4 variant mimicked. OA-NO_2_ increased DNA DSBs in irradiated EV cells (tail moment) probably because of other OA-NO_2_ targets in HR such as RAD51^11, 13^, and low endogenous RECQL4 levels in U2OS cells. This is supported by overexpressing RECQL4 WT which rescues the EV phenotype. In contrast, overexpression of the C1052S variant failed to rescue the EV phenotype. Accordingly, RECQL4 overexpression of WT protein increased OA-NO_2_ EC50 values observed in EV and C1052S cells. RECQL4 targeting by OA-NO_2_ was further supported in clonogenic survival assays, where its inhibition resulted in decreased cell survival of irradiated cells that was observed at a lesser extent in EV and C1052S cells. This suggests that OA-NO_2_ targets C1052 in RECQL4. Loss of RECQL4 by siRNA sensitizes U2OS cells^56^. Interestingly, the C1052S variant did not display a RECQL4 loss phenotype, warranting further investigation into the biological role of this residue in RECQL4 biology. One could speculate that its role could affect the interaction of the RECQL4 C-terminus with its N-terminus, thus affecting replication initiation as recently described for a RECQL4 C-terminus mutant^72^.

In contrast to a recent study, we did not find that RECQL4 WT overexpression decreased DSB formation after DNA damage^22^. This is likely due to the lower expression levels of exogenous RECQL4 proteins observed here compared to the cited study. Consistent with Luong et al^22^, however, we found no degradation of RECQL4 WT proteins after IR treatment. However, we observed a decrease in the expression of the RECQL4 C1052S mutant, suggesting that C1052 may regulate RECQL4 stability post-IR treatment.

Literature has shown that RECQL4 involves various DNA repair mechanisms beyond its role in unwinding DNA for end resection. For instance, phosphorylation by DNA-PKs enhances DNA double-strand break (DSB) repair through non-homologous end joining (NHEJ), as the phosphorylation of RECQL4 facilitates its association with Ku70/80^50^. OA-NO_2_ does not impact NHEJ^11^, raising the question of whether this is due to the effect of OA-NO_2_ on RECQL4’s phosphorylation^50^ or if it sterically inhibits the interaction between RECQL4 and NHEJ core factors. Additionally, other functions of RECQL4 have been described in suppressing RAD52-mediated single-strand annealing (SSA) pathways^73^. Given that we observed a significant inhibition of SSA by OA-NO_2_, there may be other targets of OA-NO_2_ within the SSA pathway. Future studies will be necessary to address these critical questions.

In conclusion, we provide compelling new evidence for a reversible covalent inhibitor that targets RECQL4 helicase activity in endresection and potentially will benefit patients in cancer treatment.

## MATERIALS AND METHODS

### Cell lines and cell culture

U2OS osteosarcoma cells were purchased from the American Type Culture Collection (ATCC). COV318 ovarian cancer cells were purchased from the European Collection of Authenticated Cell Cultures (ECACC). U2OS SSA-GFP and EJ2-GFP cells were a generous gift from Dr. Jeremy Stark. All cell lines were maintained in DMEM supplemented with 10% FBS (v/v) at 37 °C in 5% CO_2_. Cell line experiments involving OA-NO_2_ were performed in DMEM supplemented with 5% FBS (v/v) to prevent absorption by excess serum.

### Plasmids, oligonucleotides, and purified protein

The pCMV-Tag2B-RECQL4 plasmid was a generous gift from the laboratory of Dr. Kara Bernstein^74^, and pCMV-Tag2B-RECQL4-C1052S plasmid was generated by commercial site-directed mutagenesis services provided by Gene Universal (https://www.geneuniversal.com) using pCMV-Tag2B-RECQL4 as a template. The pCMV-Tag4A plasmid used as an empty vector (EV) control was a generous gift from Dr. Tomasz Kulikowicz. I-SceI pCAGGS plasmid for GFP reporter assays was a generous gift from Dr. Maria Jasin^75^. All transfections were performed using Fugene 6 Transfection Reagent (Promega) according to the manufacturer’s instructions in Opti-MEM media (Gibco), and experiments were conducted 48 hr post-transfection to allow for adequate transgene expression.

Fluorescently-labeled poly-T (Tiaed forked duplex oligo substrates for gel-based helicase assays were comprised of Mix 3/19-IRDye800 (labeled strand) annealed to Mix 4/19 (unlabeled strand) oligos (Integrated DNA Technologies). 3’ ssDNA tail oligo duplex substrates for real-time fluorescence-based helicase assays were composed of T3-Cy3 (top strand) annealed to B1-Dab (bottom strand) oligos (biomers.net). RECQL4^449–1111^ variant was expressed and purified from eukaryotic cells using pETM-22-RECQL4^449–1111^ cDNA as described elsewhere^35^.All oligonucleotide sequences are outlined in the table below.

### LC-MS/MS global proteomics analysis and identification of novel nitroalkene targets

U2OS cells were subjected to 4 Gy IR in a Gammacell 40 exactor γ-irradiator (Best Medical) with a dose rate of 69R/min or brought to room temperature in the case of 0 Gy controls. Immediately after IR, cells were treated with 400mM NEM (Sigma-Aldrich) or vehicle DMSO for 5 min in PBS at room temperature. This was followed by treatment with 5 μM OA-NO_2_ or vehicle DMSO in culture media and returned to the incubator. 6 hr later, cells were again alkylated with NEM to prevent thiol jumping by OA-NO_2_, collected by trypsinization, and pelleted by centrifugation.

Cell pellets were diluted in 100mM triethylammonium bicarbonate (TEAB) and sonicated. Samples were subjected to pressure-assisted digestion using a barocycler (Pressure Biosciences) and heat-stable (SMART) trypsin (ThermoFisher Scientific). Briefly, cells were transferred to Pressure Cycling Technology MicroTubes (Pressure BioSciences) for a final volume of 20 μL of 100 mM TEAB pH 8.0 with 10% acetonitrile. Samples were incubated at 99°C for 30 min, followed by 50°C for 10 min. Cells were lysed and digested by cycling 60 times between 45,000 psi for 50 s and atmospheric pressure for 10 s at a constant temperature of 50°C. Each protein digest was transferred to a clean 0.5 mL microcentrifuge tube, vacuum dried, and resuspended in 100 μL 100 mM TEAB. Peptide digest concentrations were determined using the bicinchoninic acid (BCA) assay (ThermoFisher Scientific). Peptide (40 μg) from each sample was labeled with a unique isobaric tandem mass tag (TMT) label using the TMT-11-plex Isobaric Label Reagent Set (ThermoFisher Scientific) according to the manufacturer’s instructions. A reference sample, generated by pooling equivalent amounts of peptide digests from each of the samples in the cohort, was labeled with TMT “Channel” 126. After quenching, the TMT-11 multiplex set of samples was combined and vacuum dried to approximately 80 μL.

The TMT-11 multiplex was separated offline using basic reversed-phase liquid chromatographic (bRPLC) fractionation on a 1260 Infinity II liquid chromatograph (Agilent Technologies) into 96 fractions through the development of a linear gradient of acetonitrile (0.69% acetonitrile/min) followed by concatenation into 36 pooled fractions. Each fraction’s 10% (volume) was removed for LC-MS/MS analysis. The remaining 90% (volume) of the 36 fractions was pooled into six fractions for serial phosphopeptide TiO_2_ enrichment, followed by iron-immobilized metal ion affinity chromatography (Fe-IMAC). Briefly, peptide fractions were vacuum dried, resuspended in TiO_2_ binding/equilibration buffer and bound to TiO_2_ affinity spin tips (High-Select TiO_2_ Phosphopeptide Enrichment Kit, ThermoFisher Scientific), and sample flow-through and washes were reserved for subsequent enrichment by Fe-NTA (nitrilotriacetic acid) affinity chromatography (High-Select Fe-NTA Phosphopeptide Enrichment Kit). Samples were resuspended in 0.1% formic acid for LC-MS/MS analysis.

Peptide identifications were generated against a public human non-redundant Fasta database (Swiss-Prot, downloaded 2017/12/01). The resulting peptide spectral matches (PSMs) were processed for global proteomic data with a standardized pipeline. Briefly, PSMs containing ≥ 50% isolation interference, missing data in the reference channel, or missing data in all sample channels were removed. The log2 PSM abundance was calculated for each sample channel relative to the reference before normalization by mode-centered z-score transformation of each channel. Proteins satisfying two unique peptide identifications were median aggregated. Statistical analysis was performed by comparing log2 proteomic abundances using a two-tailed z-test in R (version 4.3.1).

### Bioinformatics analyses

Pathway analysis was performed by subjecting the UniProtKB accession numbers of differentially regulated proteins as determined by the absolute log2-fold change relative to DMSO to Gene Set Enrichment Analysis (GSEA) using the freely available PANTHER GO-Slim analysis package (https://pantherdb.org). The top GO biological process annotations for upregulated and downregulated proteins in each treatment condition were plotted and visualized using GraphPad Prism v.10.

Information on RECQL4 modifications in cancer was acquired by querying publicly available TCGA data using the cBioPortal for Cancer Genomics database (https://www.cbioportal.org) and extracting information on RECQL4 modifications in relevant cancer subtypes. Data was visualized using GraphPad Prism v.10. Kaplan-Meier plots to analyze the effect of *RECQL4* expression on survival were generated and analyzed using the online survival analysis tool KM plotter (https://kmplot.com/analysis).

### CuAAC-mediated enrichment of nitroalkene targets

U2OS cells were irradiated in a Precision Cell X-Ray Irradiator (CellRad) and then treated with 5 μM Alk-OA-NO_2_ at appropriate time points post-IR. After 1 hr, treated cells were lysed on ice in tissue culture plates by scraping in CuAAC-compatible lysis buffer (50 mM HEPES pH 7.4, 100 mM sodium phosphate, 2 mM MgCl_2_, 10 U/mL DNAse I, 1% Triton X-100) supplemented with protease and phosphatase inhibitors (PPI). Lysates were sonicated, clarified by centrifugation, and diluted 1:1 in lysis buffer without Triton X-100 to reduce the detergent concentration.

CuAAC was performed directly in the diluted lysates using the following method: First, the components of the CuAAC reaction were added to the lysates in the following order and to the following concentrations: (*1*) 20 μM Diazo biotin azide in DMSO (Vector Labs), (*2*) 10 μM TBTA in DMSO (Vector Labs), (*3*) 1 mM Cu (II) SO_4_ in H_2_O (ThermoFisher Scientific), and (*4*) 3 mM Sodium L-ascorbate in H_2_O (ThermoFisher Scientific) with vortexing after the addition of each ingredient. The CuAAC reaction was allowed for 1 hr at room temperature while protected from light and quenched by adding four volumes of acetone. Protein was allowed to precipitate at −20 °C for 1 hr or overnight and then pelleted by centrifugation. The supernatant was removed, and any residual acetone was allowed to evaporate from the pellet at room temperature for 10 min.

The pellets were resuspended in a resolubilization buffer (PBS pH 7.4, 6 M Urea, 1% SDS) by sonication. 10% (volume) of resolubilized protein was kept as input control, and the rest was diluted 1:1 in PBS and incubated with pre-washed Streptavidin magnetic beads (Pierce) by rocking for 1 hr at room temperature. The beads were separated from the solution on a DynaMag magnetic rack (ThermoFisher) and washed twice with Re-solubilization buffer for two min with rocking and three times with SDS wash buffer (TBS pH 7.4, 1% SDS) for two min with rocking. The bound proteins were eluted from the beads by boiling at 95 °C for 5 min in 1X reducing Laemmli sample buffer (BioRad). An appropriate volume of eluate and input control for each sample was loaded onto an 8 or 10% Tris-Glycine gel and separated by SDS-PAGE. Separated proteins were then transferred to a nitrocellulose membrane, probed by western blot with appropriate antibodies, and bands were visualized on an Odyssey CLx imaging system (LiCor)—ImageJ quantified band intensities.

### RECQL4 structural analyses

Full-length RECQL4 domain diagrams were created using Illustrator for Biological Sequences (http://ibs.biocuckoo.org)^76^. RECQL4 residues that alter helicase activity and their respective locations were sourced from previously published data^34^. Crystal structures depicting RECQL4^449–1111^ highlighting the three-dimensional location of Cys1052 were generated as previously described^35^. The ball-and-stick model of RECQL4 and the accompanying close-up of the three-dimensional neighborhood of Cys1052 were generated using the molecular modeling software from PyMOL.

### Gel-based biochemical helicase assays

Duplex substrates were prepared by annealing Mix3/19-IRDye800 and Mix 4/19 oligos at a 1:2 ratio by boiling at 95°C for 5 min in Annealing Buffer (50 mM Tris-HCl pH 7.5, 50 mM NaCl) and allowing the oligos to cool slowly to RT for 1 hr. Purified RECQL4^449–1111^ was diluted to a concentration of 50 nM in assay buffer (25 mM Tris-HCl pH 7.5, 5 mM MgCl_2_, 10% glycerol, 1mM TCEP, 25 nM Mix 4/19, 0.1 mg/mL BSA) and aliquoted to an appropriate number of PCR tubes. ATP (Abcam) was added to the appropriate samples at a concentration of 5mM, and samples were incubated with various concentrations of OA-NO_2_ for 10 min at RT. Afterward, annealed forked substrate was added to the samples to a final concentration of 1nM to initiate the reaction and reactions and were allowed to proceed for 30 min at 37°C in a PCR System 9700 thermal cycler (GeneAmp). The helicase reactions were transferred to ice and quenched by adding 3X Stop Buffer (25 mM Tris-HCl pH 8.0, 10 mM EDTA, 1% SDS). Heat-denatured samples were boiled at 95°C for 5 min, and all reaction products were loaded onto a 10% Native TBE gel and separated by electrophoresis in 0.5X TBE (Sigma-Aldrich) at 100V for 1 hr at 4°C. Reaction products were visualized directly on an Odyssey CLx imaging system, and ImageJ quantified ssDNA production. For experiments where RECQL4^449–1111^ was pre-treated without ATP, reactions were initiated by sequentially adding substrate and 5mM ATP.

### Real-time fluorescence-based helicase assays

Short 3’ ssDNA overhang duplex substrate was prepared by annealing T3-Cy3 and B1-Dab oligos at a 1:1 ratio in 20mM HEPES annealing buffer (100mM NaCl) by boiling at 95°C for 5 min and gradually cooling the oligos to 15°C at a rate of 1°C/min in a Thermal cycler (Eppendorf). Helicase assays were conducted in a Clariostar microplate reader (BMG LABTECH) at a temperature of 25 °C in a 50 μL reaction mixture containing 100 nM RECQL4^449–1111^ pre-treated with various concentrations of OA-NO_2_ for 15 min in assay buffer (20 mM HEPES pH 8.0, 5% glycerol, 2.5% DMSO, 1 mM MgCl_2_, 2.5 mM ATP, 0.5 mM TCEP). Initially, the baseline fluorescence was recorded for 5 min, and then the reaction was initiated by adding 3’ ssDNA overhang substrate. DNA unwinding was observed by detecting an increase in the fluorescence signal due to the separation of the Dabcyl quencher and the Cy3 fluorophore. Cy3 fluorescence was measured at an excitation wavelength of 530 nm and an emission wavelength of 580 nm for 30 min. Measurements using assay buffer instead of protein served as a blank and were subtracted from the helicase data. All experiments were performed in triplicate.

### Recruitment of fluorescently tagged RECQL4 to sites of laser-induced micro-irradiation

2.5 μg of pEGFP-N-RECQL4 or pEGFP-N-MRE11 was transfected into U2OS cells using Lonza Solution V (Program X-001) according to the manufacturer’s instructions. The cells were split into two 35 mm glass bottom cell culture dishes (Mattek) with 10 μM BrdU. 24 h post-transfection, the cells were used for the micro-irradiation assays with pre-treatment of 5 μM OA-NO_2_ or DMSO for 30 min. The medium was replaced with prewarmed CO_2_-independent medium with or without OA-NO_2,_ and the plates were placed in the microscope chamber set at 37°C. Micro-irradiation was conducted with a Carl Zeiss Axiovert 200M microscope with a Plan-Apochromat 63X/NA 1.40 oil immersion objective (Carl Zeiss). A 365-nm pulsed nitrogen laser (Spectra-Physics) was set at 80% of maximum power output with the pulsed nitrogen laser to generate laser-induced DSBs. Time-lapse images were taken using an AxioCam HRm camera (Carl Zeiss). Fluorescence intensities of the micro-irradiated and control areas were measured using Carl Zeiss Axiovision software (v.4.91), and the resulting intensity of the irradiated area was normalized to the non-irradiated control area to obtain the alteration of the interested proteins.

### Quantification of nuclear staining by IF

For end resection experiments in U2OS cells, cells were plated at a density of 200,000 cells/well on glass coverslips (VWR) in 6-well tissue culture plates, and DNA was labeled by medium supplemented with 20 μM BrdU (Abcam) for 48 h and then treated with drugs as indicated. For end resection experiments in COV318 cells, cells were plated at a density of 500,000 cells/well on glass coverslips in 6-well tissue culture plates, allowed to attach overnight, and transfected the next day with appropriate plasmids. 48 h later, transfected cells were treated with drugs as indicated. For 53BP1 foci experiments, U2OS cells were plated at a density of 150,000 cells/well on glass coverslips in 6-well tissue culture plates in media containing appropriate plasmids and transfection agents. 48 h later, cells were treated with drugs, immediately irradiated, and allowed to recover for 6 h before sample processing.

Following the appropriate incubation period in all experiments, the drug was removed, and treated cells were washed twice with PBS before nuclei extraction by incubation with Pre-extraction Buffer (50 mM HEPES, 1 mM MgSO_4_, 25 mM KCl, 3 mM EGTA, 0.5% Triton X-100) at RT for 60 s. Nuclei were washed twice in PBS, formalin-fixed for 20 min at 4°C, and permeabilized with 0.5% Triton X-100 for 10 min at RT. Next, coverslips were washed in PBS and transferred to a homemade humidity chamber for incubation with IF Blocking Buffer (PBS pH 7.4, 10 μg/mL BSA, 1% goat serum) for 1 hr at RT. After blocking, nuclei were labeled overnight with appropriate primary antibodies (1:200 in 1% BSA) at 4°C, washed 4×5 min in PBS, and labeled with proper fluorescently tagged secondary antibodies (1:1000 in 1% BSA) for 1 hr at RT while protected from light. After antibody incubation, nuclei were washed for 4×5 min in PBS, mounted on glass microscope slides with DAPI (Prolong), and analyzed by fluorescence microscopy (Leica Thunder Imager) for COV318 experiments or by confocal microscopy for U2OS experiments.

For end resection experiments, RPA and/or BrdU nuclear intensity values were quantified by ImageJ, and positive nuclei were defined as nuclei with fluorescence values above an empirically determined threshold value for each independent experiment. For 53BP1 foci experiments, the number of 53BP1 foci in each nucleus was counted by eye.

### Cell cycle analysis by flow cytometry

U2OS cells were seeded at a density of 1 × 10^6^ cells/well in a six-well tissue culture plate and treated with 5μM OA or OA-NO_2_ for 30 min. Treated cells were trypsinized, collected by centrifugation, and fixed by drop-wise addition of ethanol. Cellular DNA was labeled with propidium iodide (Abcam) resuspended in FACS buffer (PBS pH 7.4, 1% FBS), and cellular DNA content was analyzed on a BD LSR II flow cytometer (BD Biosciences). The proportion of cells in each cell cycle phase was analyzed using FACS Diva software (BD Biosciences).

### Total BrdU incorporation assay

U2OS cells were seeded at a density of 200,000 cells/well in a 6-well tissue culture dish and labeled with 20 μM BrdU for 48 hr in either maintenance DMEM with 5% FBS (v/v), serum-free DMEM as a negative control, or DMEM supplemented with 10% FBS (v/v) as a positive control respectively. BrdU was removed, and cells were treated with vehicle DMSO, OA, or OA-NO_2_ for 90 min. Following drug treatment, cells were trypsinized, pelleted, and fixed in 3% paraformaldehyde (PFA) for 15 min at 4°C. Fixed cells were acid-washed in 1N HCl for 30 min to expose total BrdU and subsequently neutralized in phosphate/citric acid buffer for 30 min to prevent excessive DNA degradation. Total BrdU was labeled by sequential incubation with BrdU primary antibody (1:200 in FACS buffer) for 1 hr and fluorescently-labeled secondary antibody (1:1000 in FACS Buffer) for another hour. Cellular DNA was labeled with DAPI, and BrdU levels were quantified by flow cytometry as a function of DNA content. The total BrdU incorporation was analyzed using FACSDiva software.

### GFP reporter assays to monitor DSB repair

U2OS GFP reporter cells were plated at 200,000 cells/well in a 6-well plate, transfected with I-SceI to induce DSBs, and allowed to recover in the incubator. 6 hr later, transfection complexes were removed, and transfected cells were incubated with 5μM OA or OA-NO_2_ for 48 hr with drug replenishment 24 hr post-transfection. Treated cells were trypsinized, collected, and fixed with 3% paraformaldehyde in PBS, and GFP expression was evaluated by flow cytometry. The proportion of GFP-positive cells in each treatment condition was determined by FACSDiva software.

### Quantification of phospho Ser132-γH2AX induction.

U2OS (200,000 cells/well) or COV318 (500,000 cells/well) cells were reverse transfected with plasmids encoding WT RECQL4 protein or RQ4-C1052S mutant protein as described below. 48 hours later, transfected cells were pre-treated with 5uM OA-NO_2_ for 30 minutes as indicated, fatty acid was removed, and transfected cells were incubated with 1uM CPT for 3 hours in the case of U2OS cells, or 1 hour in the case of COV318 cells. Following drug treatment, cells were lysed in RIPA buffer (10mM Tris-HCl pH 8.0, 140mM NaCl, 1mM EDTA, 0.5mM EGTA, 1% Triton X-100, 0.1% sodium deoxycholate, 0.1% SDS) supplemented with protease and phosphatase inhibitors by scraping on ice. Lysates were sonicated, clarified by centrifugation, and protein concentration was determined by BCA. 20ug lysate/sample was loaded onto a 14% Tris-Glycine gel, separated by SDS-PAGE, and analyzed by western blot with antibodies against p-H2AX and actin as a loading control using the Odyssey CLx scanner. Normalized p-H2AX intensity was calculated using ImageJ.

### Crystal violet assay to quantify cell viability for EC50 analysis

U2OS cells were transfected with DNA by Fugene at 1:3 ratio, i.e., empty vector, wild-type RECQL4, and mutant RECQL4 C1052S. Cells were seeded in 96-well tissue culture plates at a density of 5,000 cells/well in media and transfected with DNA by Fugene at 1:3 ratio, i.e., empty vector, wild-type RECQL4, and mutant RECQL4 C1052S. Cells were then allowed to recover overnight. The next day, transfected cells were treated with proper concentrations of drugs and/or irradiated as required. 24 h later, the drug was removed, and cells were washed with PBS and fixed with formalin for 20 min at RT. Fixed cells were washed once with DI water and stained with crystal violet solution for 20 min at RT. The stained cells were washed 3x with tap water and dried overnight. Next, the crystal violet stain was dissolved in methanol for 20 min, and the absorbance value of 600nm in each well was measured using a GloMax Explorer plate reader (Promega). Cell viability was quantified as absorbance value relative to DMSO control wells. All experiments were performed in duplicate.

### Neutral Comet assay

U2OS cells were transfected with DNA by Fugene at 1:3 ratio, i.e., empty vector, wild-type RECQL4, and mutant RECQL4 C1052S. Cells (10,000) resuspended in ice-cold PBS were mixed with 150 μL of 1% low-melting-point agarose. 100 μL aliquots of this cell-agarose mixture were then spotted on glass slides previously coated with 1.2% agarose, covered with coverslips, and allowed to set at 4°C for 15 minutes. Following coverslip removal, slides were then placed in a neutral lysis solution containing 2% Sarkosyl, 0.5 M Na_2_EDTA, 0.5 mg/mL proteinase K, pH 8.0, and incubated in the dark at 4°C for 18 hours. Slides were then washed for 5 minutes each in deionized water and TBE buffer, 0.4 M Tris-HCl, 0.4 M boric acid, 10 mM EDTA. Electrophoresis was performed at 30V in freshly prepared TBE buffer for 25 minutes. Then, the slides were washed with deionized water for 5 min and stained with Ethidium Bromide (3 μg / mL) solution for 10 min, following three 5-min washings in deionized water. Fluorescence microscopy images have been acquired through a Nikon ECLIPSE Ti2 microscopy. Comet assay analysis was done using the Comet Score 2.0 software, TriTech, Sumerduck, VA, by scoring a minimum number of 100 cells per sample. Cells containing DSBs were identified based on a tail moment.

### Single-Cell Survival Assay/Clonogenic assay

The U2OS cells were transfected with DNA by Fugene at 1:3 ratio, i.e., empty vector, wild-type RECQL4, and mutant RECQL4 C1052S. There were 800 U2OS cells seeded per well in a 6-well plate, which was then incubated for 48 hours. The cells were treated with 10 μM OA-NO_2_, radiation dose of 0 and 4 Gy, or both treatments together. After exposure, the cells were maintained in an incubator for seven days. The colonies were fixed with 15% formalin for 20 minutes and stained with crystal violet for 20 minutes. Colony formation was quantified by ImageJ software.

### Statistical analyses

Unless otherwise indicated, all statistical analyses were conducted in Graph Pad Prism v. 10. Significance cutoff threshold values were set at α = 0.05. The statistical tests used to quantify significance values in each experiment are denoted in the figure legends. Multiple comparisons testing used in ANOVA analyses are performed using Tukey’s post hoc tests. EC_50_ values were calculated by fitting cell viability data to a non-linear variable slope dose-response model. Data is plotted as the mean +/− SEM unless otherwise indicated.

## Extended Data

**Extended Data 1:**
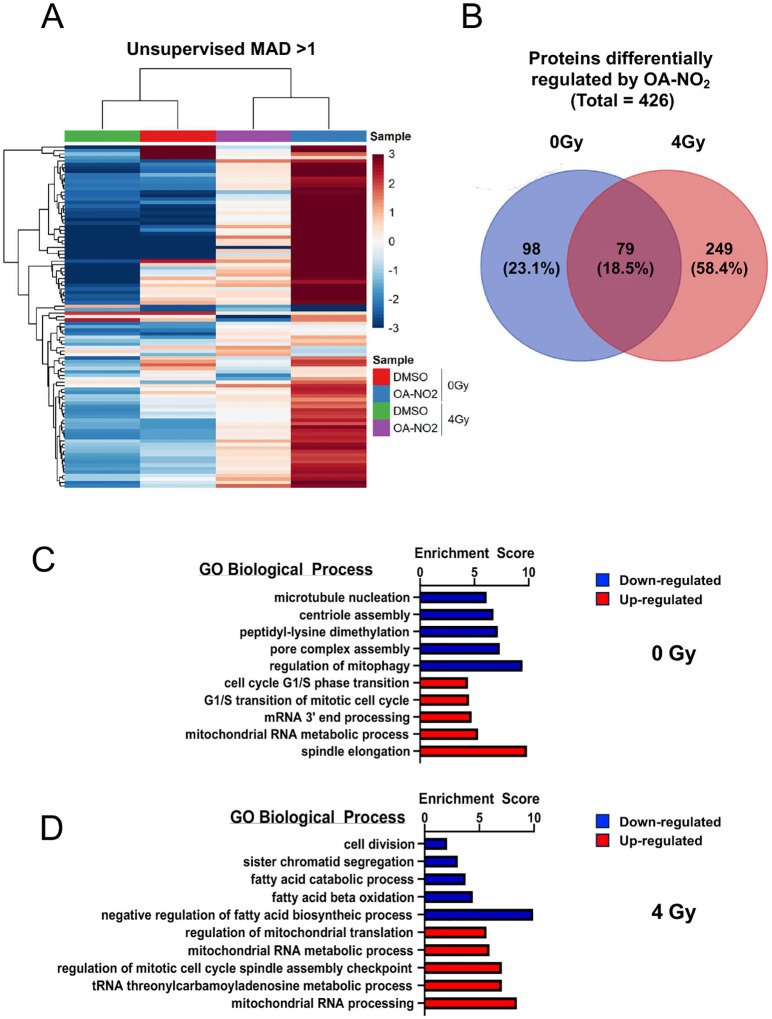
Nitroalkene modification of the cancer proteome is adaptive and dependent on the cellular redox environment. (**A**) Heat map produced by unsupervised clustering of experimental conditions based on the most significantly differentially regulated proteins identified in our analysis. (**B**) Venn Diagram comparing the overlap between proteins differentially regulated by OA-NO_2_ in the presence (4 Gy) or absence (0 Gy) of IR. (**C**) Top 5 GO biological process annotations upregulated (red bars) and downregulated (blue bars) by OA-NO_2_ in the presence of IR (0 Gy). (**D**) Top 5 GO biological process annotations upregulated (red bars) and downregulated (blue bars) by OA-NO_2_ in the absence of IR (4 Gy).

**Extended Data 2:**
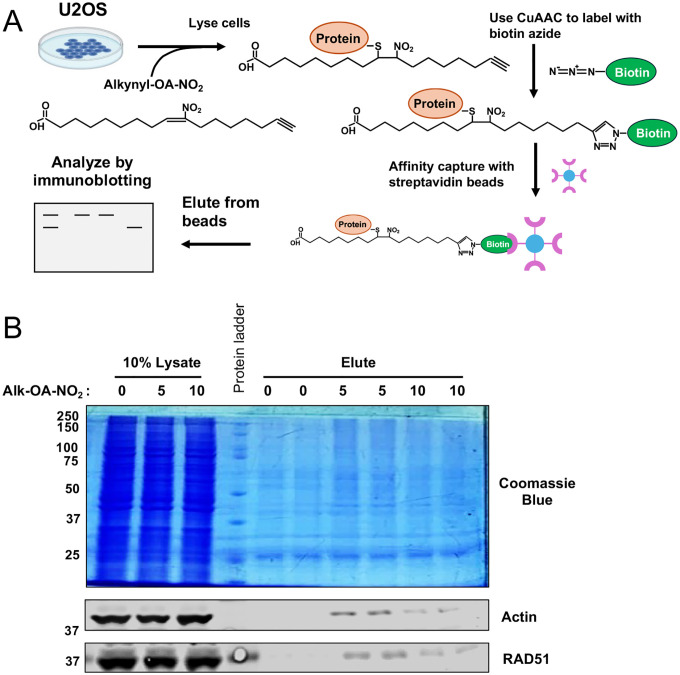
Design and validation of the CuAAC chemoproteomics approach to identify nitroalkene targets in the cancer proteome. (**A**) Schematic depicting the experimental design of the CuAAC approach to enrich for nitroalkene targets. (**B**) SDS-PAGE analysis of nitroalkene target enrichment by the CuAAC chemoproteomic approach. (Top panel) Total protein enrichment was evaluated using Coomassie Blue staining. (Bottom panel) Western blot analyzed the CuAAC-mediated enrichment of the previously validated nitroalkene targets RAD51 and β-actin.

**Extended Data 3:**
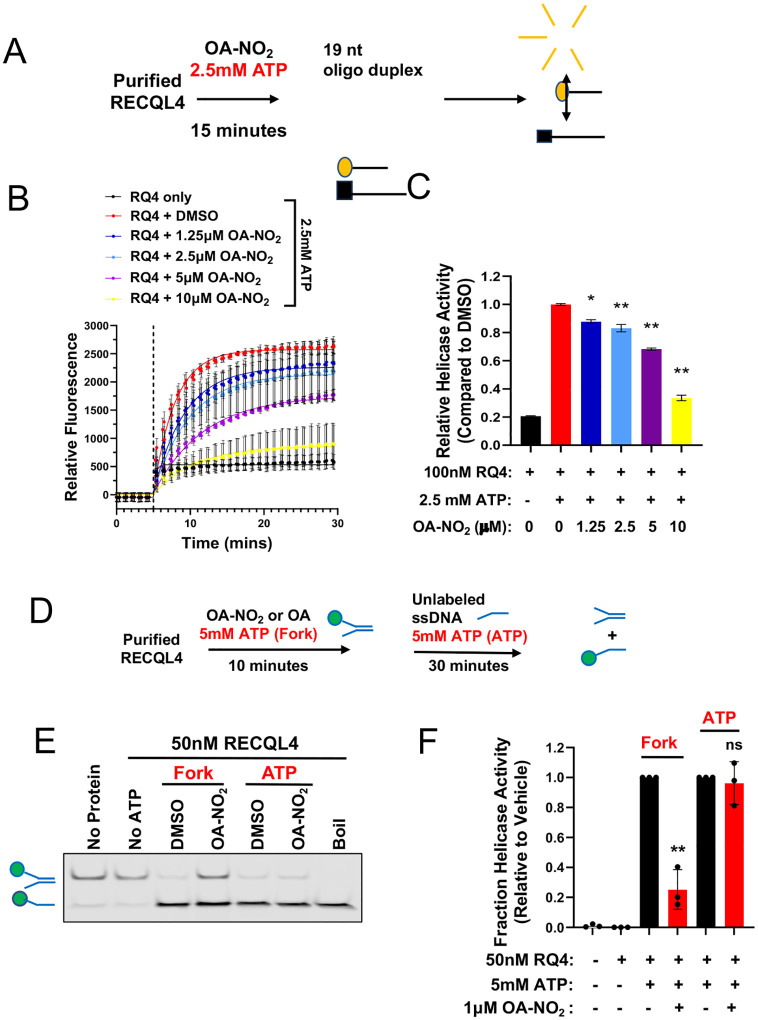
Nitro-alkylation of RECQL4 disrupts helicase activity and is dependent upon ATP. (**A**) Schematic depicting the experimental design of real-time fluorescence-based helicase assay. Substrate unwinding was measured by Cy3 fluorescence due to the increased separation of the Cy3 and Dabcyl tags on the top and bottom strands of the DNA substrate. (**B**) Quantification of helicase activity over time relative to substrate alone. Mean +/− SEM of N = 3 independent experiments. The dashed line indicates the time of substrate addition. (**C**) Maximum helicase activity was determined by fitting real-time helicase data to a one-phase association model. Mean +/− SEM of N = 3 independent experiments. Statistical significance was quantified by one-way ANOVA followed by multiple comparison testing. *, p < 0.05. **, p < 0.01. (**D**) Schematic depicting gel-based RECQL4 biochemical helicase assay. Experiments where OA-NO2 is added in the presence of ATP are denoted as Fork. Experiments where ATP is reserved until the end of the reaction are denoted ATP. (**E**) Representative blot of the effect of initiating the helicase reaction with either ATP (ATP) or substrate (Fork) on the ability of OA-NO_2_ (1 μM) to disrupt RECQL4 helicase activity. (**F**) Quantification of helicase activity as ssDNA formation relative to vehicle for ATP or substrate-initiated reactions, respectively. Mean +/− SEM of N = 3 independent experiments. Statistical significance was quantified by one-way ANOVA followed by multiple comparison testing. ns, not significant. ** p < 0.01.

**Extended Data 4:**
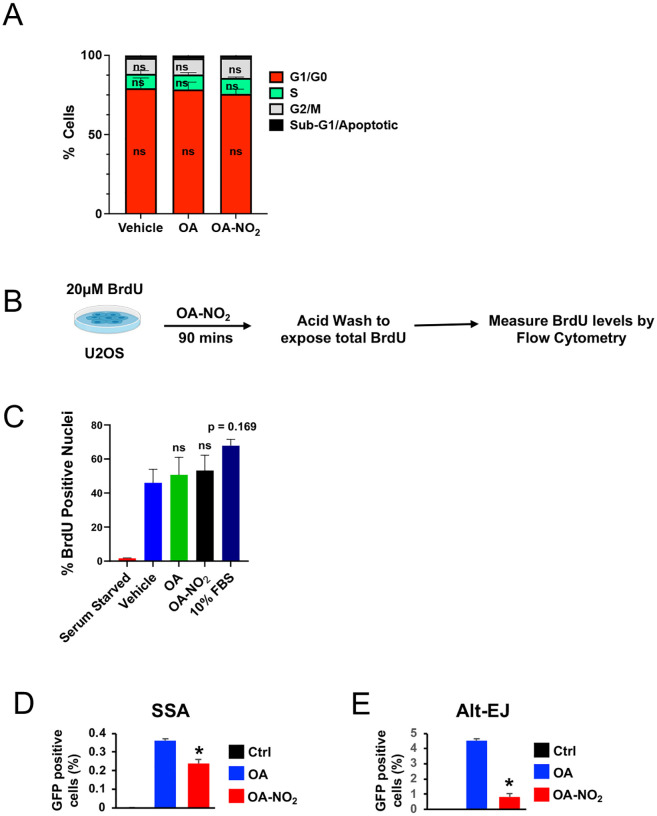
The effects of OA-NO_2_ on ssDNA formation cannot be explained by alterations to the cell cycle or BrdU incorporation. (**A**) Quantifying the effect of OA and OA-NO_2_ treatment on the proportion of U2OS cells in each cell cycle phase as measured by PI staining. Mean +/− SEM of N = 3 independent experiments. Statistical significance was calculated by two-way ANOVA followed by Tukey’s test for multiple comparisons. ns, not significant. (**B**) Schematic depicting the design of the experiment to test the effect of OA and OA-NO_2_ on total BrdU incorporation into nuclear DNA by flow cytometry. (**C**) Quantification of the effect of OA and OA-NO_2_ treatment on the percentage of BrdU-positive nuclei as measured by flow cytometry. Serum starvation and growth in enriched FBS medium (10%) were included as negative and positive controls, respectively. Mean +/− SEM of N = 2–3 independent experiments. Statistical significance was calculated by one-way ANOVA followed by Tukey’s test for multiple comparisons. ns, not significant. (**D and E**) The effect of OA-NO_2_ on fluorescence in GFP reporter cells monitoring SSA (SSA-GFP) and Alt-EJ (EJ5-GFP), respectively. Mean +/− SEM of N = 3 independent experiments. Statistical significance was calculated by one-way ANOVA followed by Tukey’s test for multiple comparisons. *, p < 0.05.

**Extended Data 5:**
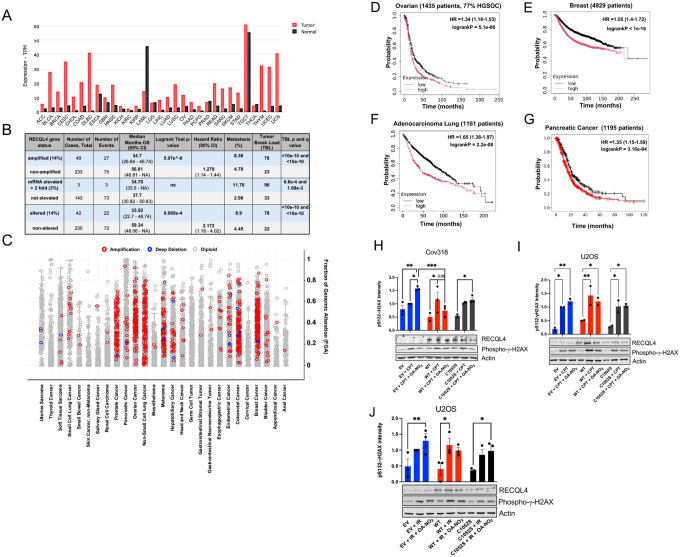
RECQL4 is an emerging pan-cancer therapeutic target. (**A**) RECQL4 transcripts per million (TPM) in 31 cancer types compared to normal tissues. Source: GePIA^41^. Abbreviations describing cancer types are available in Extended Data Table 1. (**B**) Overall patient survival (OS) based on RECQL4 mRNA or amplification from the ICGC/TCGA pan-cancer data set is depicted^42–45^. The incidence of metastasis and genomic instability were analyzed. The latter was quantified as tumor break load. This metric quantifies the level of genomic instability within a tumor sample by calculating the total number of unbalanced structural variants (SVs), like deletions and duplications, present in the tumor’s genome. Statistical analysis was performed using the Wilcoxon Test. (**C**) Quantification of RECQL4 amplifications and deep deletion in the MSK Met Tropism cohort in cbioportal^43–46^. (**D-G**) Kaplan-Meier plots analyzed by KmPlotter^47^ of the relationship between RECQL4 expression and probability of survival in ovarian, breast, lung, and gastric cancer. Hazard ratios (HR) and log-rank p values are included for each cancer subtype analyzed. (**H-J**) Immunoblot analysis of Cov318 and U2OS cells expressing RECQL4 WT or the C1052S variant. Cells were treated with 10 μM OA-NO_2_ before treatment with CPT (1 μM) or IR (10 Gy). Protein bands were quantified by ImageJ and first normalized to actin of the individual experiment and then to EV 10 Gy. Cells were harvested after 3 h for cell lysis.

**Extended data Table 1,:** TCGA used abbreviations for cancer types.

TCGA	Detail
ACC	Adrenocortical carcinoma
BLCA	Bladder Urothelial Carcinoma
BRCA	Breast invasive carcinoma Cervical squamous cell carcinoma and endocervical
CESC	adenocarcinoma
CHOL	Cholangial carcinoma
COAD	Colon adenocarcinoma
DLBC	Lymphoid Neoplasm Diffuse Large B-cell Lymphoma
ESCA	Esophageal carcinoma
GBM	Glioblastoma multiforme
HNSC	Head and Neck squamous cell carcinoma
KICH	Kidney Chromophobe
KIRC	Kidney renal clear cell carcinoma
KIRP	Kidney renal papillary cell carcinoma
LAML	Acute Myeloid Leukemia
LGG	Brain Lower Grade Glioma
LIHC	Liver hepatocellular carcinoma
LUAD	Lung adenocarcinoma
LUSC	Lung squamous cell carcinoma
MESO	Mesothelioma
OV	Ovarian serous cystadenocarcinoma
PAAD	Pancreatic adenocarcinoma
PCPG	Pheochromocytoma and Paraganglioma
PRAD	Prostate adenocarcinoma
READ	Rectum adenocarcinoma
SARC	Sarcoma
SKCM	Skin Cutaneous Melanoma
STAD	Stomach adenocarcinoma
TGCT	Testicular Germ Cell Tumors
THCA	Thyroid carcinoma
THYM	Thymoma
UCEC	Uterine Corpus Endometrial Carcinoma
UCS	Uterine Carcinosarcoma
UVM	Uveal Melanoma

## Figures and Tables

**Table 1:** List of OA-NO_2_ peptides identified.

UniProtKB Identifier	Full Protein Name	Gene name	Alkylated Fragment	Biological Process
**Q9UL36**	Zinc finger protein 236	ZN236	**QAELQDEPKHANCCTYCPK**	Transcription regulation
**P04085**	Platelet-derived growth factor subunit A	PDGFA	**CTGCCNTSSVK**	Growth factor
**Q8TCN5**	Zinc finger protein 507	ZN507	**ISSLAPPSMEYCVLLFCCCICGFESTSK**	Transcription regulation
**Q86UZ6**	Zinc finger and BTB domain-containing protein 46	ZTB46	**HGVCTDCAGR**	Transcription regulation
**Q99496; Q06587**	E3 ubiquitin protein ligase RING2	RING2	**SLHSELMCPICLDMLK**	Transcription regulation
**Q6NX45**	Zinc finger protein 544	ZN544	**ILTHQRIHTGEKPYQCIECGK**	Transcription regulation
**P13671**	Complement component C6	CO6	**GEVLDNSFTGGICK**	Complement activation
**P83436**	Conserved oligomeric Golgi complex subunit 7	COG7	**YLSDSCSPRSLAGFQESILTDK**	Protein transport
**Q13355**	Probable methyltransferase TARBP1	TARB1	**DVIHCTMITHQILLR**	tRNA methylation
**Q15884**	Protein FAM189A2	F1892	**ILVARFLEQSSCTMTPDIHELVENIK**	Unclear
**Q15911**	Zinc finger homeobox protein 3	ZFHX3	**MEGCDSPVVSGK**	Transcription regulation
**Q5JXX5**	Glycine receptor subunit alpha-4	GLRA4	**ITLILSCLMDLK**	Unclear
**Q6NSI3**	Protein FAM53A	FA53A	**SLSEPEELVRCR**	Protein transport into nucleus
**O94761**	ATP-dependent helicase-like Q4	RECQL4	**DQICDFLYGR**	DNA replication and repair
**Q8IVV7**	Glucose-induced degradation protein 4 homolog	GID4	**DISGASFAGFYYICFQK**	Proteasomal degradation
**Q8WWZ1**	Interleukin-1 family member 10	IL1FA	**CSLPMARYYIIK**	Cytokine, inflammation
**Q8WWZ7**	ATP-binding cassette subfamily A member 5	ABCA5	**ILLLDEPTAGMDPCSR**	Cellular transport
**Q96BT3**	Centromere protein T	CENPT	**ALEMVEKCLDK**	Cell cycle, cell division, Mitosis
**Q96N46**	Tetratricopeptide repeat protein 14	TTC14	**AIEDFELALENCPTHR**	Ubiquitin conjugation
**Q9C0D7**	Probable ribonucleotide ZC3H12C	ZC12C	**IPYCGMPQDPPR**	RNA regulation
**Q9P0V3**	SH3 domain-binding protein 4	SH3B4	**SCSISPVLEVK**	Endocytosis

**Table 2:** Oligonucleotide sequences for DNA substrates.

Oligo	Label	Sequence (5’ - 3’)
Mix 3/19- IRDye800	5’ IRDye800CWN	TTTTTTTTTTTTTTTGAGTGTGGTGTACATGCAC
Mix 4/19	None	GTGCATGTACACCACACTCTTTTTTTTTTTTTTT
T3-Cy3	5’ Cy3	CCATTCCACCCTCTATTTTTTTTTTTTTTT
B1-Dab	3’ Dabcyl	TAGAGGGTGGAATGG
